# Cecal epiploica appendix torsion in a female child mimicking acute appendicitis: a case report

**DOI:** 10.1186/1757-1626-2-8023

**Published:** 2009-05-29

**Authors:** Efstratios Christianakis, Nikolaos Paschalidis, Georgios Filippou, Dimitrios Smailis, Maria Chorti, Spiros Rizos, Dimitrios Filippou

**Affiliations:** 1 Pendeli Children’s HospitalPalaia Pendeli, AthensGreece; 2 First Department of General Surgery, Pireaus General Hospital “Tzaneio”Piraeus, AthensGreece; 3 Department of Pathology, Sismanoglion General HospitalBrilissia, AthensGreece; 4 Department of Anatomy, Nursing Faculty, University of AthensAthensGreece

## Abstract

Acute appendicitis is the most common cause of the right lower quadrant acute abdominal pain in children. Some other conditions including cecal epiploica appendix torsion, can simulate acute abdomen. Epiploica appendix torsion usually occurs in the sigmoid colon and rarely in the cecum of adult males. In children, this entity is extremely rare and may represent a diagnostic and therapeutic dilemma. We report a case of an 8-year-old Greek girl, presented with signs and symptoms mimicking acute abdomen. Our patient is the younger one among the other four with cecal epiploica appendix torsion that had been reported in the literature.

## Introduction

Torsion and infarction of appendiceal epiploicae may rarely be found in adults of any age that are treated for acute abdominal pain, although it is very rare in younger children. The majority of epiploica appendix torsion cases occur in the sigmoid colon of middle aged men suffering from acute abdominal pain of the left lower abdomen. A correct preoperative diagnosis is essential because the patient can be treated conservatively to avoid an unnecessary operation; although many authors suggest that the definitive treatment of epiplocae appendicae torsion should be the surgical excision.

## Case presentation

**Figure 1.**
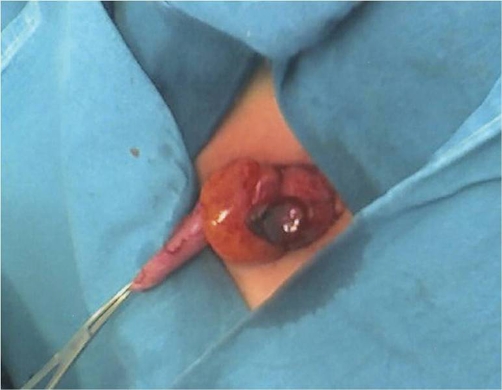
Cecal appendix epiploica with torsion and necrosis in a child. Vermiform appendix looks without inflammation; the cecum has excessive fat tissue and many large epiploic appendices, arranged in two separate longitudinal rows.

**Figure 2.**
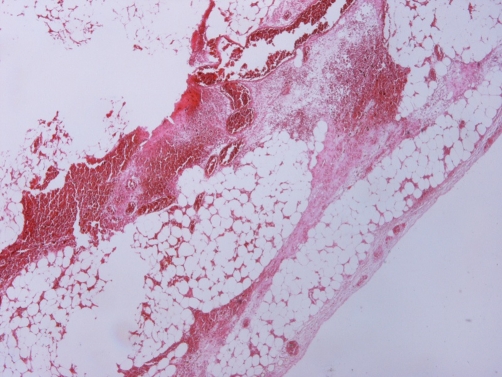
Histological examination showed ischemic infarct of the appendix epiploica adipose tissue.

An 8-year-old Greek girl presented with acute abdominal pain in the right lower quadrant that begun ten hours before. The patient claimed that was feeling bad and suffering from localized abdominal guarding, bad appetite and nausea without vomiting. On physical examination, the patient felt uncomfortable even at rest. The cardiac rythms was 85 beats/min, while the arterial blood pressure of 105/68 mmHg. Abdominal tenderness localized to the right lower quadrant was noted. The patient’s temperature was normal and the rectal examination failed to reveal any pathology. The white blood cell count was 13,000/mm^3^ with 61% neutrophils, and 26% lymphocytes. Hemoglobin was 12.4 g/dL, hematocrit 37.5% and platelets 348,000. Urine and blood biochemical examinations were normal. Ultrasonography did not reveal any vermiform appendix or solid hyperechoic mass, but revealed some fluid in the right iliac fossa and in Douglas’ space. Six hours later, the abdominal pain worsened. The patient’s general condition also worsened. We repeated the abdominal ultrasound but there was no evidence of an intra-abdominal pathology. The patient operated on an emergency basis for acute abdomen and an ischemic, twisted, 4 cm long appendix epiploica in cecum was found (Figure 1). The vermiform appendix did not inflammed, but the entire large intestine and particularly the sigmoid colon had excessive fat tissue and many large epiploicae appendices. A small quantity of serous-bloody fluid was aspirated. Appendectomy and ligation with removal of the appendix epiploica were performed. The histological examination of the specimen revealed an ischemic infarct of the appendix epiploica adipose tissue, while the histological examination of the veniform appendix was normal (Figure 2). The pain immediately disappeared postoperatively and the patient was discharged the third postoperative day, without presenting any postoperative complications.

## Discussion

Epiplocae appendicae are pendulant adipose structures protruding from the serosal surface of the large intestine that are arranged in two separate longitudinal rows. Each one of them is supplied by one or two small end arteries branching from the long rectum vessels of the colon and is drained by a tortuous vein passing through its narrow pedicle. Their limited blood supply, together with their peduncle shape and excessive mobility, make them prone to spontaneous torsion and ischemic or hemorrhagic infarction [1]. In 1908, Briggs first reported a case of an epiploca appendix torsion (appendagitis) mimicking appendicitis [2]. Disease of the epiplocae appendicae is presented with localized abdominal pain with or without peritoneal signs, minimal fever and leukocytosis [3,4]. Epiplocae appendicae may infract and necrosed as a result of torsion or venous thrombosis. The inflammatory reaction that follows acute torsion or spontaneous thrombosis of the draining vein and this process called primary epiploic appendagitis or primary appendicitis epiploica [5]. Epiplocae appendicae are affected also by calcification due to aseptic fat necrosis, pericolic abscess, enlargement by lipomas or metastases, and incarceration in hernias.[3] The clinical presentation of an acute torsion can also mimic diverticulitis or cholocystitis [4]. Epiplocae appendicae torsion can rarely be seen in patients younger than 19 years and is almost unknown in children. The symptoms usually resolve within 1 week (mean, 4.7 days) without surgical treatment. Although computed tomography findings (mainly a mass of adipose tissue) will completely resolve in 3-6 months after the initial acute presentation, the disease may recurrent mimicking acute abdomen [5,6]. A recent search of the literature from the authors revealed four similar cases in children [3,7,8,9].

Acute abdomen is the main cause of acute abdominal pain in children, although in some rare cases atypical cases can be found. There are no ultrasonographic descriptions of cecal epiploica appendix torsion in children and only one from four reported cases with children had a non-operative diagnosis by computed tomography. This imaging technique is not used in children with acute abdomen routinely, except in very doubtful and severe cases. These observations suggest that cecal epiploica appendix torsion in childhood will probably continue be difficult to set preoperatively and most of the patients will be diagnosed intraoperatively. Furthermore, in cases with cecal epiploica appendix torsion that are diagnosed preoperatively by imaging techniques, a prolonged period of the symptoms, probably due to partial torsion may be an indication for surgical treatment.

The radiological findings of cecal epiploica appendix torsion in adults have been well described in the literature as predictable and consistent. Ultrasound may reveal a solid, well-delineated non-compressible hyperechoic mass, adjacent to the colonic wall. Spotty color areas with arterial flow into this mass can be identified by acolor Doppler sonograph. Detection of abnormalities of the colonic wall adjacent to infiltrated fatty tissue and absence of flow may suggest the diagnosis [3]. Computer tomography usually reveal a circumscribed oval fatty mass representing the infracted or inflamed epiploica appendix, with hyper attenuating streaks surrounding the mass that represents the inflamed visceral peritoneal lining, all of which are usually adherent to the serosal colonic surface [2]. In selected cases a central high attenuation dot and a lobulated fatty mass, representing engorged or thrombosed central vessels or central areas of bleeding can be seen. Para-colonic inflammatory changes are disproportionally more severe than a mild local reactive thickening of the adjacent colonic wall [3]. Magnetic resonance findings include an oval shaped fat intensity mass with a central dot on T1- and T2-weighted images, which posses an enhancing rim on postgadolinium T1-weighted fat saturated images [3].

Nowadays, laparoscopy may represent a valuable tool and an acceptable therapeutic alternative for both diagnosis and treatment of cecal epiploica appendix torsion. Although there are many author’s who support that definitive treatment of in adults is surgical excision; conservative treatment with antibiotics and analgesics seems also to be safe.

In conclusion, torsion, necrosis, or inflammation of an epiploica appendix of the large bowel may present a diagnostic and therapeutic dilemma requiring surgical intervention [10].
